# Societal costs and effects of implementing population-based mammography screening in Greenland

**DOI:** 10.1080/22423982.2017.1373580

**Published:** 2017-09-22

**Authors:** Maria Klitgaard Christensen, Birgit V. Niclasen, Kim Moesgaard Iburg

**Affiliations:** ^a^ Department of Public Health, Aarhus University, Aarhus, Denmark; ^b^ Department of Health, Chief Medical Consultant, Nuuk, Greenland; ^c^ Department of Public Health, Aarhus University, Aarhus, Denmark

**Keywords:** Economic evaluation, mammography screening, breast cancer, cost-effectiveness analysis, cost-utility analysis, Greenland

## Abstract

With a low breast cancer incidence and low population density, Greenland is geographically and organisationally challenged in implementing a cost effective breast cancer screening programme where a large proportion of the Greenlandic women will have to travel far to attend. The aim of this paper is to evaluate the cost effectiveness and cost utility of different strategies for implementing population-based breast cancer screening in Greenland. Two strategies were evaluated: Centralised screening in the capital Nuuk and decentralised screening in the five municipal regions of Greenland. A cost effectiveness and cost utility analysis were performed from a societal perspective to estimate the costs per years of life saved and per QALY gained. Two accommodation models for the women’s attendance were examined; accommodation in ordinary hotels or in patient hotels. The least costly accommodation model was the hotel model compared with the patient hotel model, regardless of screening strategy. The decentralised strategy was more cost effective compared with the centralised strategy, resulting in 0.5 million DKK per years of life saved (YLS) and 4.1 million DKK per quality-adjusted life year (QALY) gained within the hotel model. These ratios are significantly higher compared with findings from other countries. The sensitivity analysis showed a substantial gap between the most and least favourable model assumptions. The investigated strategies were all estimated to be extremely costly, mostly due to high transportation and accommodation costs and loss of productivity, and none would be accepted as cost-effective per YLS/QALY gained within a conventional threshold level. The least expensive strategy was regional screening with hotel accommodation.

## Introduction

Breast cancer is the second most common type of cancer among women in Greenland and constitutes 14% of all cancer cases diagnosed and 7% of every cancer death every year among Greenlandic women [1]. The Nordic countries, Canada and Alaska in the US, which Greenland usually look to for comparison, have implemented organised breast cancer screening with mammography to reduce the breast cancer mortality [2]. Currently, a screening programme does not exist in Greenland. Greenland usually follow the World Health Organisation’s guidelines, which states, for the introduction of a national screening programme, that the economic costs should be balanced fairly in respect of the health utility gained [3]. Despite being the largest island in the world, Greenland has only around 56,000 inhabitants living scattered on the 2,000 km long coastline in about 16 small towns and 60 villages [4]. The extremely low population density, together with the country’s limited and time consuming transportation options are huge infrastructural challenges to the health sector [5] and would, presumably, have an influence on the cost effectiveness of an implementation of a screening programme. The awareness of the adverse effects screening entail has been highlighted elsewhere and negative factors such as over-diagnosis, false positives and psychological distress influence whether the benefits of screening outweigh the harms [6]. The cost effectiveness of a breast cancer screening programme is influenced also by contextual factors in the healthcare system, the healthcare costs, the chosen screening interval, the chosen age interval of the women invited and the breast cancer epidemiology [7]. The magnitude of the effect of breast cancer screening is still disputed [8–11]. A relative risk reduction (RRR) in breast cancer mortality of 20% in women invited to screening compared with non-invited control groups was found by a UK panel and Cochrane, both only including RCT-studies in their systematic review [6,9]. Two systematic reviews over incidence based studies estimated a 25–26% RRR [10,11], while a review of quasi-experimental studies found an effect of 16–23%, depending on the type of study design [8]. Consequently, the expected screening effect on breast cancer mortality is attached with uncertainty.

This paper seeks to evaluate the costs and consequences of different strategies for an implementation of population-based breast cancer screening in Greenland from a societal perspective.

## Methods

### Screening strategies

The strategies compared in this evaluation were (1) breast cancer screening and triple testing in the capital, Nuuk and (2) breast cancer screening in the five regional hospitals in Greenland, but with triple testing in Nuuk. Within both strategies, women aged 50–69 years would be offered biennial screening in accordance with the European screening guidelines [2]. In Greenland, the health system bears the full travel and accommodation costs of all citizens attending the services for national screening programmes. The women who accept the screening offer would be flown or sailed to either the National Hospital in Nuuk or to the closest city with a regional hospital, depending on which city is closest to the woman’s home. Two models of accommodation while the women await their travel back home were examined in both strategies; accommodation in either an ordinary hotel or in a patient hotel. In the centralised strategy, there are two permanent radiographers who could conduct the mammograms all year, while the regional strategy would require the radiographers to travel to the other four cities with a regional hospital and conduct the mammograms over a couple of weeks. In both strategies, the mammograms would be sent to Denmark for clinical assessment. Unforeseen expenditures were assumed to be an additional 15% of the yearly costs in the centralised strategy and an additional 20% in the regional strategy after advice from the Department of Health. Other alternative screening strategies were investigated, but were rejected since they were not considered to be possible to implement due to practical issues.

### Data collection

A systematic literature search about the screening effect on breast cancer mortality or quality-of-life was made in the PubMed database from September 2014 until January 2016. National and international websites [12–15] and relevant articles’ reference lists were also searched. Estimates of the RRR in breast cancer mortality and quality-of-life weights were chosen based on the literature search. Information about the self-reported health status of Greenlandic women in the age interval 50–69 years was provided from the national health survey from 2005–2009, which is based on a stratified sample of the adult population in Greenland [16]. One general health question with five categories was used to measure self-reported health [16].

Registry data about population size and income was stratified by home town and collected together with a life table from Statistics Greenland [17]. Data about breast cancer diagnosis, birth year, age at diagnosis and breast cancer deaths in Greenland from the years 2004–2013 was provided by the Office of the Chief Medical Officer and statistics on the incidence and mortality in Greenland was collected from the database NORDCAN [1]. Data related to costs of health services were collected from the Agency of Health and Prevention in Greenland. This includes standard prices on facilities, mammography equipment, breast cancer operation, chemotherapy, accommodation in hotel or patient hotel and administration fares. Health personal agreements were identified for their respective wages. The travel costs and time was mainly provided by Air Greenland or found on the homepage for the shipping companies, Arctic Umiaq Line or Disco Line. In the few cases where the companies did not sail or fly from a town, standard fares from the Agency of Health and Prevention were used as averages.

### The cost analysis

The cost analysis included healthcare costs, travel and accommodation costs and production costs. The software used was Excel 2007.

### Health costs

In Greenland, breast cancer cases are usually found locally by biopsy and the treatment procedure is currently mastectomy followed by chemotherapy. It was assumed that the cases found by screening would be treatable locally. Treatment costs due to over-diagnosed cases were estimated based on a 1–10% over-diagnosis rate of all lives saved [18], together with the price of mastectomy with following chemotherapy and the strategy’s average transportation and accommodation costs. Wages to healthcare personnel were estimated using their monthly salary and the length of employment per year. The standard prices on office rental, reading of mammograms, equipment and installation, education, audit and false positives were used to estimate the costs related to facilities and diagnostic. Resources saved due to fewer biopsies were estimated based on a standard price/biopsy, the current number of biopsies/year and the attendance rate.

### Travel and accommodation costs

In both strategies, 70% of the women invited were assumed to attend after advice from the Agency of Health and Prevention. There is no empiric evidence from the health sector, suggesting a different attendance rate between going to the regional cities or to the capital in a Greenlandic setting, since a considerable part of the population has to travel far to attend either way and without bearing the monetary costs. However, a lower participation in the centralised strategy is also examined in the sensitivity analysis. The travel costs were estimated using specific round-trip ticket fares, including administration costs, together with the residence distribution of women aged 50–69 years. If it was possible to travel both by plane and ship, the alternative with the lowest costs was chosen. Two accommodation models were examined; accommodation in an ordinary hotel or in a patient hotel. The average numbers of nights away depending on home town and the fares for one night including meals were used to estimate the accommodation costs in the two models. Currently, there is no excess capacity in the patient hotels in Greenland, so costs of expanding the existing patient hotels were also included in this model. The expansion costs in the two screening strategies were based on the estimated number of beds needed per patient hospital, together with the standard area per bed, including joint surroundings and the price per square metre. The yearly maintenance costs were estimated based on a standard price per square metre given by Agency of Health and Prevention in Greenland.

### Production loss

The production loss was estimated using the human capital approach and the average gross daily income for Greenlandic women aged 50–69 years, stratified by region and town size, together with the number of women resident and their days away from home to participate in the screening programme.

### Breast cancer screening effect

The breast cancer screening effect was assumed to be independent of screening place and, therefore, the same in both strategies. The absolute number of lives saved was estimated on the basis of the method used by Hendrick & Helvie [19] to estimate the number of women needed to invite to save one life. Table 1 shows the assumptions and values used. The number of breast cancer deaths per 1,000 women was estimated using the cumulated breast cancer mortality for Greenlandic women aged 50–80 years [17]. The cumulated risk was adjusted for time of diagnosis, since women diagnosed with breast cancer before age 50 can inherently not benefit from a screening invitation after age 50. The RRR in breast cancer mortality because of invitation to mammography screening was assumed to be 20% on the basis of the literature search [6,8–11]. The absolute number of lives saved during a 10-year screening and follow-up period were estimated using the total number of women aged 50–69 years in Greenland from 2013.

**Table 1.** Values in the screening strategies.

Parameter	Estimate	Comments
Number of women in the age interval 50–69 years resident in Greenland	5,812 women	Based on the year 2013 from Statistics of Greenland.
Screening age	50–69 years	Recommended age interval for mammography screening in Europe [2].
Screening interval	2 years	Recommended screening interval for mammography screening in Europe [2].
Screening and follow-up period (screening rounds)	10 years (5 rounds)	Chosen from the screening and follow-up periods in the scientific articles examining the screening effect.
Maximal age at follow-up	79 years	Benefits and harms can occur until the maximal age, which should, therefore, be included in the analysis.
Cumulated breast cancer mortality for Greenlandic women aged 50–80 years	2.14%	The estimate was based on the years 2004–2013 and is extracted from NORDCAN [1]. Because of data availability, the upper age limit is 80 instead of 79 years.

The average health expectancy was estimated using Sullivan’s [20] method. For women aged 50–69 years, the self-reported health status data was used to estimate the average quality-of-life weight (QoL) for the norm population and a life table was used for the calculation of the average number of years of life saved (YLS). To calculate the quality adjusted life years (QALY’s) gained, the absolute number of lives saved was adjusted using the QoL and YLS for the target population. Adverse screening consequences were included by adjusting for the reduction in QALY’s due to over-diagnosis and false positive results. The QoL weights and the time spent in each health state were chosen on the basis of the literature search [21,22]. The reduction was estimated using 0.81 QoL for a false positive test result, 0.48 for a mastectomy and 0.74 for chemotherapy [21] over a 6-month period [22] for both false positives and over-diagnosis. The number of women with a false positive test result during the 10-year period was estimated based on a Danish false positive rate of 2.8% [23] and the number of over-diagnosed cases was estimated using an estimate of 1–10% of over-diagnosed cases of all the lives saved [18]. To obtain the absolute number of QALY’s gained during the period, the number of QALY’s lost due to adverse effects were deducted from the QALY’s gained due to screening.

### Cost effectiveness and cost utility analysis

In the cost effectiveness analysis, the total costs per strategy were divided by the years of life saved to estimate the costs per YLS. In the cost utility analysis, the costs per QALY gained were estimated. In both analyses, the costs and utilities have been discounted with 0% and 3% over the 10-year period. The model parameters attached with most uncertainty were the RRR, the needed screening and follow-up period and the attendance rate, which were examined using the most extreme values in the sensitivity analysis. The percentage of women needed to attend and the needed number of screening rounds required to reach a certain RRR would presumably have a substantial influence on the total costs because of the high travel and accommodation costs in Greenland. By assuming a high RRR in breast cancer mortality of 26% after only 6 years of screening with a low 50% attendance rate, the most beneficial values were examined and, by assuming a low RRR of 15% after 20 years with a maximum of 100% attendance, the least beneficial values were estimated. Furthermore, a 10% lower attendance rate in the centralised strategy compared with the regional was investigated using 50% vs 60% and 60% vs 70%.

## Results

### Costs

After 10 years with regional screening, the total costs were significantly lower compared to centralised screening in Nuuk, respectively, 149.4 million DKK and 255.9 million DKK, including unforeseen costs in the hotel model. The lower costs with decentralised screening were mainly attributed to the lower production loss and travel costs per screening round. The same tendency was not found in the patient hotel model where decentralised screening resulted in a total cost of 297.6 million DKK after 10 years compared with 263.3 million DKK with centralised screening (Table 2). The higher expenses related to the expansion of the patient hotels in the regional strategy, respectively 78.2 million DKK against 25.8 million DKK for centralised screening, were not compensated over the 10-year period by the lower production loss and travel costs per year.

**Table 2.** Overview of society costs (DKK).

	Screening in Nuuk			Regional screening		
Category	Unit costs	Yearly costs	Costs after 10 years	Unit costs	Yearly costs	Costs after 10 years
*Society costs*						
Production loss	980	2,962,427	29,624,268	801	2,328,725	23,287,252
*Health sector costs*						
Transportation costs						
Travel costs*^a^*	4,959	10,016,902	100,169,017	3,817	3,179,831	31,798,309
Hotel accommodation*^b^*	1,100	3,387,410	33,874,097	1,100	2,671,477	26,714,765
Patient hotel accommodation*^b^*	675	2,078,638	20,786,378	675	1,639,315	16,393,151
Patient hotel*^c^*	—	1,984,000	25,792,000	52,459,384	6,014,000	52,459,384
Staff, equipment and facilities						
Radiographer*^d^* (salary/month)	26,018	811,746	8,117,463	62,530	562,770	5,627,700
Education of radiographer and audit	230,000	70,000	900,000	230,000	70,000	900,000
Medical secretary*^d^* (salary/month)	19,733	1,231,352	12,313,517	19,733	923,514	9,235,138
Office (furnished)	45,000	120,000	360,000	45,000	30,000	315,000
Evaluation consultant	—	468,000	4,680,000	—	468,000	4,680,000
Mammograph	1,500,000	60,000	2,100,000	1,500,000	150,000	6,000,000
Mammograph transportation, installation and facility renovation	360,000	50,000	860,000	840,000	150,000	2,100,000
Reading of mammograms*^e^*	232,500	152,750	1,677,500	232,500	182,500	1,825,000
Mastectomy*^f^*	22,180	2,218	22,180	22,180	2,218	22,180
Chemotherapy*^f^*	78,549	7,855	78,549	78,549	7,855	78,549
						
*Total costs* (hotel model)		17,749,333	177,508,327		9,834,139	104,356,392
*Total costs* (patient hotel model)		16,440,561	180,811,558		8,801,978	146,494,163

An attendance rate of 70% was assumed. The costs after 10 years of screening are inclusive of establishment costs.

*^a^*Average ticket price t/r including taxes, fees and potential accommodation on the journey.

*^b^*Accommodation costs, including food.

*^c^*Establishment and yearly maintaining costs for expanding the patient hotels in the five regions.

*^d^*Including 30% of the salary for recruiting & retention and education & audit.

*^e^*Establishment of PACS-system and external reading of 2,900 mammograms per year.

*^f^*Costs are assumed to by divided equally throughout the years, since the time of occurrence for over-diagnosed cases is unknown.

### Screening effect

The absolute number of lives saved was estimated to 16 women with a RRR of 20% in breast cancer mortality. By varying the RRR to 10 and 26%, the absolute number of lives saved ranged from 8–21 lives over the 10-year period. The proportion of women aged 50–69 years with a good self-reported health was 0.57 and the average number of YLS were 19.2. Adjusting the absolute number of lives saved with the QoL weight and YLS resulted in a gain of 171 QALYs. With an attendance rate of 70% in both strategies, 133 QALYs were lost due to false positive results and over-diagnosis. This resulted in an absolute number of 38 QALYs gained over the screening and follow-up period. By varying the RRR in breast cancer mortality to 10% and 26%, the respective results were a loss of 48 QALYs and a gain of 91 QALYs after adjusting for negative effects. Consequently, it is possible that the harms of screening can outweigh the benefits depending on the RRR.

### Cost effectiveness and cost utility analysis

The cost effectiveness ratio in the regional strategy was 0.2 million DKK/YLS lower than the centralised strategy in the hotel model and 0.1 million DKK/YLS higher in the patient hotel model (Table 3). The same tendency was found in the cost utility (CU)-analysis. In the regional strategy, the cost utility ratio (CUR) was 2.0 million DKK/QALY gained lower in the hotel model compared with the centralised strategy and 0.9 million DKK/QALY gained higher in the patient hotel model (Table 3). The regional screening strategy was, therefore, more cost effective per YLS and QALY gained when compared with centralised screening with accommodation in regular hotels and less cost effective with accommodation in patient hotels. Of the two accommodation models, the CE- and CU-ratios were smaller in the hotel model compared with the corresponding estimates in the patient hotel model. For comprehensiveness, both costs and effects were discounted with a 0% and 3% discount rate (Table 3). The discounted results did not differ in interpretation from the undiscounted results.

**Table 3.** Cost effectiveness and cost utility analysis (DKK).

Screening strategy	Costs after 10 years	YLS	CER	QALYs	CUR
*Hotel model*					
1. Nuuk	226,127,955 (198,995,370)	308 (229)	734,945 (868,975)	38 (28)	5,950,736 (7,106,978)
2. Regional	149,378,252 (132,270,126)	308 (229)	485,499 (577,599)	38 (28)	3,931,007 (4,723,933)
*Patient hotel model*					
1. Nuuk	263,553,878 (235,248,692)	308 (229)	856,584 (1,028,291)	38 (28)	6,935,628 (8,409,953)
2. Regional	297,578,716 (273,213,699)	308 (229)	967,170 (1,193,073)	38 (28)	7,831,019 (9,757,632)

Results are shown with a 20% RRR, an attendance rate of 70% in both strategies and with 0% discounting and 3% in brackets.

The sensitivity analysis was based on the hotel model, since this was the most cost effective accommodation model. The analysis showed a wide range between the least and most favourable model assumptions, ranging from 0.2–3.5 million DKK/YLS and −5.1–0.5 million DKK/QALY (Table 4). It should be noted that the lower limit of the CU-ratios was negative, resulting in a loss in QALYs.

**Table 4.** Sensitivity analysis.

Screening strategy	Costs at follow-up (DKK)	YLS	CER	QALY’s	CUR
*Best case*					
Nuuk*^a^*	104,044,568 (96,939,233)	404 (346)	257,644 (280,058)	119 (66)	877,542 (1,476,701)
Regional*^a^*	75,747,856 (71,033,161)	404 (346)	187,574 (205,215)	119 (66)	638,879 (1,082,067)
*Worst case*					
Nuuk*^b^*	612,766,221 (470,105,685)	154 (115)	3,983,140 (4,074,412)	−105 (−88)	−5,849,126 (−5,358,152)
Regional*^b^*	376,048,840 (290,098,669)	154 (115)	2,444,415 (2,514,289)	−105 (−88)	−3,589,554 (–3,306,475)
*Attendance rate^c^*	**Δ Cost atfollow- up (DKK)**		**Δ CE**		**Δ CU**
50% vs 60%	37,141,689	308	120,715	38	−84,114
60% vs 70%	49,141,590	308	159,717	38	−460,380

The sensitivity analysis is based on the hotel model. Results are shown with 0% discounting and 3% in brackets.

*^a^*Assumptions: A relative risk reduction in breast cancer mortality of 26% after 6 years with a 50% attendance rate.

*^b^*Assumptions: A relative risk reduction in breast cancer mortality of 10% after 20 years with a 100% attendance rate.

*^c^*The estimation formula: (the centralised strategy) – (the regional strategy).

With a 10% lower attendance rate in Nuuk, regional screening was still more cost effective per YLS. In the CUA, on the other hand, regional screening was less cost effective compared with screening in Nuuk, 0.1–0.5 million less effective per QALY (Table 4).

## Discussion

### Comparison of study results

The cost effectiveness of mammography screening programmes has been examined in many countries, but this is the first study in the context of Greenland. Compared with other studies, the estimated CE- and CU-ratios are significantly higher in Greenland. A study by de Gelder *et al*. [24] of the Swiss population-based breast cancer screening programme found a cost effectiveness ratio of 11,500 Euro per YLS and a retrospective study of the American programme estimated the costs to be 24,700 Euro per QALY gained [25]. In China, an estimate of CE- and CU-ratios of ~ 58,900 Euro per YLS and 56,400 Euro per QALY gained were concluded [26]. In contrast, the most favourable option found in this paper was regional screening with accommodation in regular hotels, which encompassed 0.5 million DKK (67,500 Euro)/YLS and 3.9 million DKK (0,53 million Euro)/QALY gained. A systematic review of 17 national data sets found mammography screening cost-effective in western countries, while not in most Asian countries [27]. The authors concluded it was because of the low breast cancer incidence and ethnic characteristics in Asia [27]. The inhabitants of Greenland originate from Asia [4], which can explain the low incidence rate. The low breast cancer incidence and extremely low population density in Greenland compared with Western countries [1] entailed significant travel and accommodation costs related to the women’s attendance. These are the major reasons for the substantially low cost-effectiveness of screening compared with the programmes in other countries. Only under the most favourable model assumptions were the CU-ratios comparable with the results by Wong *et al*. [26], where the authors concluded that the scarce resources in society might be better used elsewhere.

Furthermore, the benefit of breast cancer screening is highly debated. A Swiss medical board recommended in 2014 that the country’s screening programme in the long run should be abolished because of the lack of clear evidence of the overall positive screening consequences [28]. In contrast, an independent English panel and Canadian board recommended a continuation of the countries’ programmes and concluded that the benefits outweigh the harms [6,29]; however, the Canadian board emphasised that the evidence was weak. Based on the results of this paper, the authors do not recommend implementing the investigated strategies in Greenland from a social economic perspective. The results do not live up to the mentioned guidelines of WHO regarding the introduction of a national screening programme. The scarce resources in Greenland should instead be used where the health utility gained is balanced fairly regarding the economic costs. In addition, without clear evidence that the benefit of screening outweighs the harms, it can be argued from an ethical perspective whether there is a legitimate reason to impose the distress and possible negatives consequences on the attending women.

In 2014, the Department of Health in Greenland recommended that a social economic evaluation was made to investigate the consequences of implementing population-based mammography screening in view of competing priorities. This paper has contributed with such an evaluation with the purpose to inform policy-makers. Consequently, the results of this study are of great importance for the women in Greenland and their prospect to participate in mammography screening if the study’s recommendation is followed.

### Strengths and weaknesses

The selected strategies for this study were identified in collaboration with the Board of Health and Department of Health in Greenland to ensure they were accustomed to the specific Greenlandic setting. Consequently, the study results are context-specific and cannot be used to inform the policy debate in other populations. Alternative strategies were investigated, but were rejected due to practical feasibility implications. The substantial uncertainty surrounding the screening effect on the breast cancer mortality and the QoL weights used in the estimation of the expected screening utility is a significant limitation, but this study tried to accommodate it through the sensitivity analysis. A strength in the current paper is its level of detail of the cost analysis regarding establishment of the facilities, healthcare treatment, office rental, wages, travel costs and savings due to fewer biopsies obtained from the Board of Health and the travel companies in Greenland. A last and important weakness is that the study is made on several model assumptions, which were shown in the sensitivity analysis to be crucial for the variation of the strategies’ cost effectiveness. However, the final ranking of the two strategies was not altered and under none of the assumptions would the strategies investigated be accepted as cost effective within conventional international threshold criteria.

Future studies could investigate the cost-effectiveness of other alternatives to mammography screening, such as clinical breast examination.

## Conclusion

The results suggest that an implementation of a population-based breast cancer screening programme in Greenland will have a substantially lower cost effectiveness than the screening programmes in other countries. The most favourable screening strategy was screening at the regional hospitals, with accommodation of the women in regular hotels, compared with screening only at the national hospital in Nuuk.

## Keypoints

- Regular hotel accommodation was more cost effective compared to patient hotel accommodation.
- The most favourable strategy was regional screening compared with centralised screening with accommodation in regular hotels.
- A breast cancer screening programme in Greenland will be significantly less cost effective compared with programmes in other countries.
- None of the strategies investigated would be accepted as cost effective within conventional threshold levels.
